# A new species of *Diaphorocera* from Morocco with unclear relationships and a key to the species (Coleoptera, Meloidae, Cerocomini)

**DOI:** 10.3897/zookeys.748.22176

**Published:** 2018-04-04

**Authors:** Ladislav Černý, Marco A. Bologna

**Affiliations:** 1 The South Bohemian Museum in České Budějovice, Dukelská 1, CZ-370 51 České Budějovice, Czech Republic; 2 Dipartimento di Scienze, Università Roma Tre, Viale G. Marconi 446, 00146 Roma, Italy

**Keywords:** Coleoptera, *Diaphorocera neglecta* sp. n., key, relationships, Sahara

## Abstract

*Diaphorocera neglecta* **sp. n.** from Morocco is described. Photos of the new species are provided and male features are figured. The new species has intermediate characters between the groups of *D. hemprichi* and *D. promelaena* as defined in the literature. These groups are discussed and a new key to the species is presented.

## Introduction

*Diaphorocera* Heyden, 1863 is a Saharo-Sindian genus, most diverse in the western Sahara Desert and belonging to the tribe Cerocomini, family Meloidae. The phylogenetic relationships within the tribe were defined on the basis of both morphological and molecular evidence by Turco and Bologna (2008), who pointed out the affinities of *Diaphorocera* with the eastern African genus *Somalarthrocera* Turco & Bologna, 2008. The genus was revised by Turco and Bologna (2007) who also summarised the distribution and bionomics of each species.

*Diaphorocera* is characterised by a metallic green-blue body, but with legs, antennae and mouthparts being yellow-orange in most species. Male antennae are extremely modified, as in other genera of the tribe and composed of eleven antennomeres. Turco and Bologna (2007) considered *Diaphorocera* and the genus *Anisarthrocera* as primitive elements within the tribe, both being strictly adapted to eremic habitats.

After the descriptions of some species in the XIX century, Kocher (1954) described *D. peyerimhoffi* from Morocco, while Kaszab (1983) described *D. johnsoni* and *D. hemprichi saudita*, both from the eastern part of the Arabian Peninsula. Subsequently, Batelka (2004) redescribed *D. carinicollis* Chobaut, 1921 from eastern Algeria and south Tunisia. In their revision, Turco and Bologna (2007), examined types of all taxa, synonymised *D. kerimii* Faimaire, 1875 with *D. chrysoprasis* Fairmaire, 1863, and redescribed the eighth species.

The aims of this paper are to describe a new species from eastern Morocco, *D. neglecta* sp. n., the ninth species in the genus, showing intermediate characters between the groups of *D. hemprichi* Heyden, 1863 and *D. promelaena* Fairmaire, 1876 , as defined in the literature and to design a new key to the species.

## Materials and methods

Available specimens were examined using stereomicroscopes and measurements were taken with an ocular grid. The total length of the examined specimens is measured from the anterior apex of mandibles to the apex of elytra. Proportions of antennomeres were taken on holotype male measurements. Male genitalia were dissected and glued onto a paper label.

Photographs were takenby an Olympus OM-D E-M5 camera equipped with a macro lens Olympus M.Zuiko Digital ED 60 mm f/2.8 Macro.The type specimens are labelled with a printed red rectangular label: “HOLOTYPUS or PARATYPUS respectively, *Diaphorocera neglecta* sp. n., M. A. Bologna & L. Černý des. 2016”. All specimens are glued onto paper labels.

Examined specimens are preserved in the following collections:

**CB** M.A. Bologna collection, Museum of the Department of Sciences, University Roma Tre, Roma, Italy;

**JMBC** The South Bohemian Museum in České Budějovice, Dukelská 1, CZ-370 51 České Budějovice, Czech Republic;

**LCCC** L. Černý collection, České Budějovice, Czech Republic.

## Taxonomic part

### Diaphorocera neglecta sp. n.

Taxon classificationAnimaliaColeopteraMeloidae

http://zoobank.org/F63001C5-17E1-44C4-B39A-29C1E65D4EDA

#### Type material.

Holotype ♂ (CB), Paratype ♂ (LCCC), Paratypes ♀♀ (CB, JMBC), all labelled Morocco SE, W of Erfoud, 31°30'53,2"N, 004°35'12"W, 26.–27.4.2012, L. Černý lgt. [printed].

#### Type locality.

Eastern Morocco, W of Erfoud, 31°30'53,2"N, 004°35'12"W, 26.–27.4.2012.

#### Diagnosis.

A *Diaphorocera* species characterized by male elongate last antennomere and simple fore tibiae, with head, pronotum and ventral parts dark shiny metallic blue.

**Figure 1–2.**
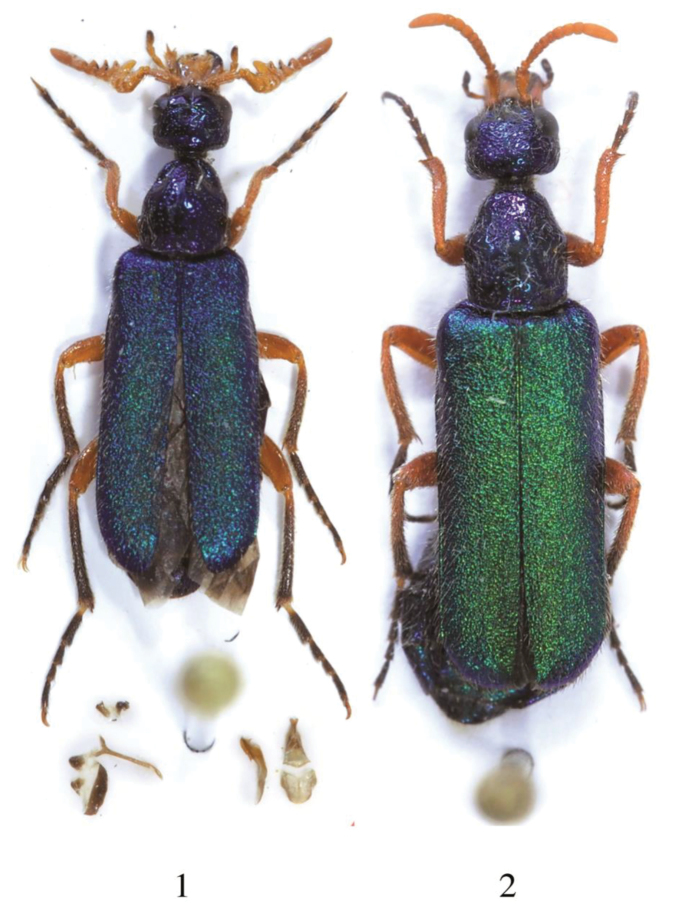
*Diphorocera neglecta* sp. n., **1** holotype male and **2** paratype female.

**Figures 3–6.**
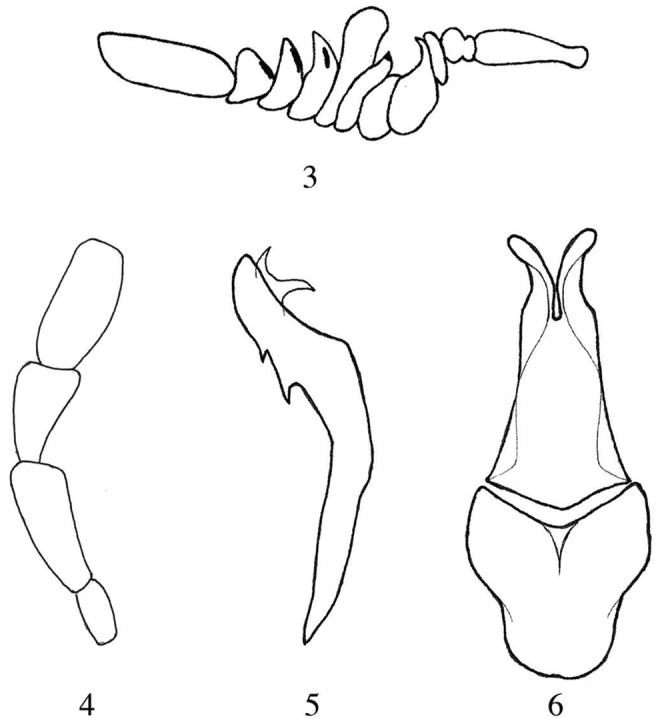
*Diphorocera neglecta* sp. n., **3** male antenna **4** male maxillary palpomeri **5** male aedeagus in lateral view **6** gonoforceps and gonocoxal piece in ventral view.

**Description.** *Male* (Fig. 1). *Body.* Head, pronotum, thorax, abdomen distinctly dark blue metallic, elytra green-blue metallic; coxae, trochanters, femurs, tibiae, and tarsi orange, but metatarsi darker and mesotarsi slightly darkened. Body and leg setation clear yellowish and in some parts almost white. Length: 9.8–9.9 mm. *Head* with punctures sparse, wide, and deep, surface among punctures smooth and shiny. Maximum width at level of eyes and temples, temple parallel, as long as the eye. Frontal callus smooth, neither keeled nor anteriorly protruding, in lateral view only few convex. Antennae as in Fig. 3; antennomeres I–XI orange, VIII–X with a single, linear and short black stripe on the apical portion of each antennomere, middle in position on VIII-IX, more basal on X; the extreme pointed apex of IV and VI black; XI very elongate, 2.8 times long as wide; X ca. 2/3 as long as XI. Mouthparts orange except the apical third of mandibles black; maxillary palpomere as in Fig. 4, last palpomere dark, as labial palpomeres; labrum ca. 3 times as long as clypeus and with a middle furrow dorsally. *Pronotum* narrow and parallel on the basal half, distinctly longer than wide, anteriorly restricted and with two lateral grooves on sides. Surface with punctures similar to those on the head, sparse but deep, surface among punctures smooth, shiny, and a longitudinal non-punctured area in the middle extending from the base to the anterior third of the length. Elytra with surface densely and coarsely punctuated, punctures merging together. Foretibiae simple and not modified; metatibial outer spur widened and spoon-like. *Male genitalia* as in Figs 5–6; aedeagus inclined on fore third, with two short and pointed hooks, hooks of the endophallic duct bent differently; lobes of parameres diverting and obtuse apically.

*Female* (Fig. 2). *Body* colouration similar to male. Antennae unmodified, length of antennomere XI equal to that of previous three together, antennomeres III–X transverse, wider than long (see Fig. 2). Lack lateral grooves of pronotum. Protibiae distinctly pointed and acuminate on external fore angle. Length: 12.5 mm.

#### Etymology.

Following the recent revision of the genera (Turco and Bologna 2007) and due to the rarity of this *Diaphorocera* species, we named it *neglecta* (= neglected, ignored) according to the Latin adjective (feminine in the nominative case).

#### Systematic remarks.

Turco and Bologna (2007) established three groups of species within the genus based on a cladistic analysis: (i) the *obscuritarsis* group (*carinicollis*, *johnsoni*, *obscuritarsis*) with the last male antennomere subquadrate and unmodified simple foretibiae; (ii) the *promelaena* group (*promelaena*) characterised by the last antennomere elongate, male foretibia modified only distally and by black body and pronotum; (iii) the *hemprichi* group (*chrysoprasis*, *hemprichi*, *sicardi*, *peyerimhoffi*) with the last antennomere elongate and foretibiae modified.

The new species differs from all described species due to the combination of characters mixed between those of the *promelaena* and *hemprichi* groups. *D. neglecta* has the last antennomere elongate as in both groups but the fore tibiae are simple, differently than in these groups; both these characters represent a plesiotypic condition. It is distinct from *D. promelaena* because its head, pronotum and ventral parts are dark metallic blue and not sub-opaque black. Moreover its pronotum is slender, longer than wide rather than as long as wide, and the black ornamentations on antennomeres VIII-X being lacking in *D. promelaena*.

*Diaphorocera neglecta* is more similar to the species of the *hemprichi* group because of the body colouration, the shape of pronotum and antennae, and probably represent a basal element without foretibial modification. It differs from all species of this heterogeneous group because of the shape of almost all antennomeres and of male genitalia; in particular the ventral shape of gonoforceps are slightly similar to that of *D. hemprichi*. The ornamentation on antennomeres is present only in *D. sicardi*, but differs greatly from that of this species which moreover is clearly distinguished by its foretibial shape.

Tentatively the new species is inserted in the group of *hemprichi*, as basal in position, although its relationship needs to be tested by molecular analysis and the possible relationships with the group of *D. promelaena* explored.

#### Ecological remarks.

All specimens were collected in a small area with many plants in bloom at the bottom of a dry river bed (wadi) at 872 m a.s.l., together with other species of Meloidae, namely *Diaphorocera promelaena*, Mylabris (Ammabris) myrmidon Marseul, 1870, *Hycleus saharicus* (Chobaut, 1901), *H. novemdecimpunctatus* (A.G. Olivier, 1811), *Actenodia suturifera* (Pic, 1896), *Croscherichia litigiosa* (Chevrolat, 1840), *Lyttolydulus nubeculosus* Kaszab, 1952, *Cabalia rufiventris* (Walker, 1871) and *Lydomophus* sp. (*chanzyi* (Fairmaire, 1876) ?), all typical Saharan elements.

A second attempt to collect further specimens of this species in the same locality (on 22 April 2017) failed, probably due to much drier conditions.

#### Distribution.

**Figure 7.**
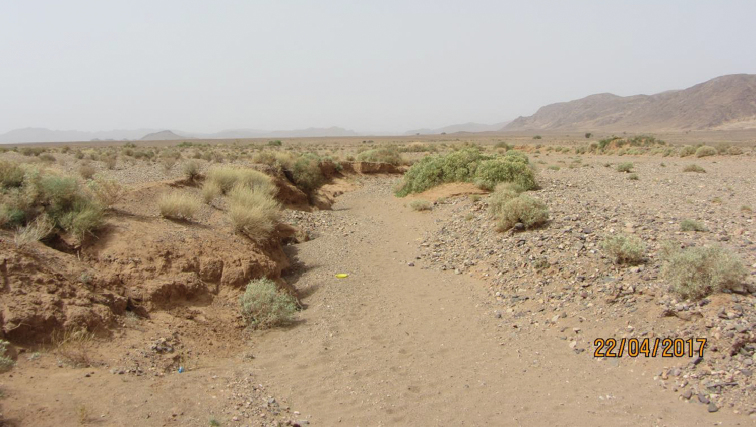
Habitat of the type locality of *Diaphorocera neglecta* sp. n.

The new species was collected only in the type locality (Fig. 7), but since the local desert environment is widely and homogeneously spread, its occurrence in other eremic habitats of the eastern Morocco is possible.

In E Morocco and SW Algeria, in localities close to Erfoud, six species of *Diaphorocera* are distributed (Turco and Bologna 2007); *D. promelaena* is syntopic with the new species. No differences in the ecological niche among these six species have been identified in the literature, all of them being synchronic and polyphagous.

##### Key to the *Diaphorocera* species (modified from Turco and Bologna 2007)

**Male**

**Table:** 

1	Antennomere XI subquadrate. Foretibiae simple	**2**
–	Antennomere XI elongate. Foretibiae variously modified or simple in one species	**4**
2	Two black and shiny lines on antennomere XI and one on antennomere X	***D. obscuritarsis***
–	Antennomeres X-XI without lines	**3**
3	Antennomere VII distinctly wider than VI and slightly narrower than VIII; antennomere I dark. Pronotum slender, anterior portion distinctly narrower than temples; anterior grooves only weakly developed. External margin of elytra only slightly sinuate	***D. johnsoni***
–	Antennomere VII ca. 1/3 wider than VI and as wide as VIII; antennomere I yellow. Pronotum robust, anterior portion only slightly narrower than temples; anterior grooves deep. External margin of elytra posteriorly greatly sinuate	***D. carinicollis***
4	Head, pronotum, abdomen and antennomere I black	***D. promelaena***
–	Head, pronotum, abdomen and antennomere I not black	**5**
5	Frontal calli with a dorsal keel anteriorly protruded and pointed; fore tibiae with a basal inflated expansion	**6**
–	Frontal calli neither keeled nor anteriorly protruding; fore tibiae simple or with a laminar expansion, at least on the external side	**7**
6	Antennomeres VIII-XI with black lines and spots, distal half of X wide, ca twice as wide as that of IX, VII obliquely truncate at apex, incision of distal portion of IV narrow	***D. sicardi***
–	Antennomeres VIII-XI without black lines and spots, distal half of X narrow, as wide as that of IX, VII bilobed at apex, incision of distal portion of IV wide	***D. peyerimhoffi***
7	Fore tibiae simple. Antennomere X only 1/3 as wide as the length of XI	***D. neglecta***
–	Fore tibiae greatly modified. Antennomere X wider than 1/3 the length of XI	**8**
8	Antennomere X about as wide as the length of XI, anterior portion slender and pointed at apex; IX about as wide as VIII	***D. hemprichi***
–	Antennomere X distinctly narrower than the length of XI. Anterior portion of antennomere IX wide and apically truncate; IX narrower than VIII	***D. chrysoprasis***
**Female**
1	Antennomere XI subquadrate	**2**
–	Antennomere XI elongate	**4**
2	Fore and middle tarsomeres IV-V dark, basal segments yellow; trochanters only slightly dark	***D. carinicollis***
–	Tarsomeres and trochanters dark, or fore tarsomere I light at base	**3**
3	Labrum completely dark; temples slightly diverging posteriad, maximum width of head on temples. Femurs and tibiae orange-red	***D. obscuritarsis***
–	Labrum dark with the anterior margin orange; temples parallel, maximum width of head on eyes. Femurs and tibiae yellow	***D. johnsoni***
4	Head and pronotum black	***D. promelaena***
–	Head and pronotum metallic	**5**
5	Coxae and trochanters black	***D. peyerimhoffi***
–	Coxae metallic, green or bluish; trochanters yellow	**6**
6	Body blue, but fore coxae yellow	***D. sicardi***
–	Body and fore coxae green or green-blue metallic	**7**
7	Head capsule transverse, about as wide as long; antennomere XI more than three times as long as the width of X	***D. neglecta***
–	Head capsule slender, longer than wide; antennomere XI less than three times as long as the width of X	**8**
8	Temples elongate, about as long as the eye length, with subparallel sides	***D. chrysoprasis***
–	Temples shorter than the eye length, narrowing evenly posteriad	**9**
9	Pronotum distinctly narrow. Head and pronotum punctures usually sparse and shallow, surface among punctures shiny, almost smooth. Temples in dorsal view squared and slightly longer; in lateral view the edge between vertex and occiput sharper. Antennomere VIII ca. as long as wide and more squared	***D. hemprichi saudita***
–	Pronotum parallel but less narrow. Head and pronotum punctures usually dense and deep, surface among punctures shagreened. Temples in dorsal view more rounded and shorter; in lateral view the edge between vertex and occiput more rounded. Antennomere VIII wider than long and more trapezoidal	***D. hemprichi hemprichi***

## Supplementary Material

XML Treatment for Diaphorocera neglecta
